# Improving a Mobile Telepresence Robot for People With Alzheimer Disease and Related Dementias: Semistructured Interviews With Stakeholders

**DOI:** 10.2196/32322

**Published:** 2022-05-03

**Authors:** Marlena H Shin, Jaye McLaren, Alvin Ramsey, Jennifer L Sullivan, Lauren Moo

**Affiliations:** 1 Center for Healthcare Organization and Implementation Research Veterans Affairs Boston Healthcare System Boston, MA United States; 2 New England Geriatric Research Education and Clinical Center Veterans Affairs Bedford Healthcare System Bedford, MA United States; 3 Pilltrax Systems LLC Boston, MA United States; 4 Center of Innovation in Long-Term Services and Supports Veterans Affairs Providence Healthcare System Providence, RI United States; 5 School of Public Health Brown University Providence, RI United States; 6 Center for Healthcare Organization and Implementation Research Veterans Affairs Bedford Healthcare System Bedford, MA United States; 7 Harvard Medical School Boston, MA United States; 8 Department of Neurology Massachusetts General Hospital Boston, MA United States

**Keywords:** mild cognitive impairment, socially assistive robot, robot technology, caregiver support, gerontology, aging in place, qualitative research, mobile phone

## Abstract

**Background:**

By 2050, nearly 13 million Americans will have Alzheimer disease and related dementias (ADRD), with most of those with ADRD or mild cognitive impairment (MCI) receiving home care. Mobile telepresence robots may allow persons with MCI or ADRD to remain living independently at home and ease the burden of caregiving. The goal of this study was to identify how an existing mobile telepresence robot can be enhanced to support at-home care of people with MCI or ADRD through key stakeholder input.

**Objective:**

The specific aims were to assess what applications should be integrated into the robot to further support the independence of individuals with MCI or ADRD and understand stakeholders’ overall opinions about the robot.

**Methods:**

We conducted in-person interviews with 21 stakeholders, including 6 people aged >50 years with MCI or ADRD living in the community, 9 family caregivers of people with MCI or ADRD, and 6 clinicians who work with the ADRD population. Interview questions about the robot focused on technology use, design and functionality, future applications to incorporate, and overall opinions. We conducted a thematic analysis of the data obtained and assessed the patterns within and across stakeholder groups using a matrix analysis technique.

**Results:**

Overall, most stakeholders across groups felt positively about the robot’s ability to support individuals with MCI or ADRD and decrease caregiver burden. Most ADRD stakeholders felt that the greatest benefits would be receiving help in emergency cases and having fewer in-person visits to the doctor’s office. Caregivers and clinicians also noted that remote video communication with their family members using the robot was valuable. Adding voice commands and 1-touch lifesaving or help buttons to the robot were the top suggestions offered by the stakeholders. The 4 types of applications that were suggested included health-related alerts; reminders; smart-home–related applications; and social, entertainment, or well-being applications. Stakeholders across groups liked the robot’s mobility, size, interactive connection, and communication abilities. However, stakeholders raised concerns about their physical stability and size for individuals living in smaller, cluttered spaces; screen quality for those with visual impairments; and privacy or data security.

**Conclusions:**

Although stakeholders generally expressed positive opinions about the robot, additional adaptations were suggested to strengthen functionality. Adding applications and making improvements to the design may help mitigate concerns and better support individuals with ADRD to live independently in the community. As the number of individuals living with ADRD in the United States increases, mobile telepresence robots are a promising way to support them and their caregivers. Engaging all 3 stakeholder groups in the development of these robots is a critical first step in ensuring that the technology matches their needs. Integrating the feedback obtained from our stakeholders and evaluating their effectiveness will be important next steps in adapting telepresence robots.

## Introduction

### Background

More than 6 million Americans aged ≥65 years are currently live with Alzheimer disease and related dementias (ADRD), and it is projected that this number will increase to nearly 13 million Americans aged ≥65 by 2050 [1]. Approximately 80% of people with ADRD and most people with mild cognitive impairment (MCI) receive care in their homes [2]. In the United States, 83% of care provided to older adults is from family members, friends, or other unpaid caregivers; 48% of these caregivers provide help to older adults with ADRD [1]. Caregivers of people with ADRD can often be burdened emotionally and financially; they experience emotional, financial, and physical challenges due to care responsibilities at twice the rate of caregivers of older adults without dementia [1]. The primary reason family members or friends act as caregivers for people with MCI or ADRD is to allow them to remain living in the community rather than in a long-term care facility, but this may become challenging, especially for caregivers supporting people with ADRD who live alone. ADRD caregivers report worsening of their own health due to care responsibilities and are required to take a leave of absence from work or quit work [3].

Various types of robots can help people with MCI or ADRD. Mobile telepresence robots, which are the focus of this study, can be used to mediate communication and social exchange between family members, friends, and others who may not be colocated. Such contact can alleviate loneliness, support medication compliance and other important daily activities, and alert caregivers and clinicians in a timely way to address changing patient needs. Studies have established the feasibility of mobile telepresence robots for supporting social interactions between caregivers and people with MCI or ADRD [4]. If telepresence is complemented by autonomous robotics, the potential range of benefits expands even further through innovative approaches to care that can allow people with MCI or ADRD to remain living independently in the community, which may help to alleviate loneliness and enhance the quality of life of people with ADRD, as well as ease the burden of caregiving [5-11].

Although several types of robots have been developed to assist people with MCI or ADRD, and the feasibility of telepresence for supporting social interactions between caregivers and people with dementia has been established [4], research on dementia-specific adaptations and acceptance of these robots that incorporate perspectives across 3 key end-user stakeholder groups (ie, individuals with MCI or ADRD, caregivers, and clinicians) has been limited in scope [4,5,11-14]. For example, there is limited research on how autonomous robotics features can assist a mobile telepresence robot in supporting at-home care of people with MCI or mild to moderate ADRD. Robots have often been developed in an ad hoc manner by technology companies with limited understanding of the needs, preferences, and feedback from key stakeholders [15-17]. The ways in which robots can assist people with MCI or ADRD, their caregivers, and clinicians need to be explored further.

### Objectives

In this study, we engaged these 3 key stakeholder groups in the design of dementia-specific adaptations to an existing, commercially available mobile telepresence robot that is already being used in several other settings, such as in schools for homebound students and for home health care by remote clinicians. The overall purpose of this qualitative study was to obtain stakeholder feedback and identify how an existing, commercially available mobile telepresence robot can be enhanced to support at-home care of people with MCI or mild to moderate ADRD, with the ultimate goal of developing an autonomous mobile telepresence robot. The specific aims of the study were to (1) assess what applications should be integrated into the robot to support the independence of individuals with MCI or ADRD and (2) understand stakeholders’ overall opinions about the robot.

## Methods

### Overview

The study consisted of qualitative interviews with 3 key stakeholder groups (people with MCI or ADRD, caregivers, and clinicians) to obtain feedback on how to adapt an available mobile telepresence robot and further understand stakeholders’ opinions about the potential utility of the robot.

### Ethics Approval

The study protocol was approved by the Institutional Review Board at the Veterans Affairs (VA) Bedford Healthcare System (approval number 110818). Participation in the study was voluntary, and informed consent was obtained from each participant.

### Study Population

Study inclusion criteria consisted of the following for each of the three stakeholder groups, respectively: (1) male or female Veteran aged ≥50 years with MCI or early ADRD living in the community (ie, not a nursing home or assisted living facility): people with MCI or ADRD were categorized as having MCI versus early ADRD based on the most recent clinic visit note at the time of recruitment and had to be alone at home for at least 4 hours, and there had to be no indication of incompetence in the Veteran’s medical record; (2) male or female family caregivers aged ≥18 years who had a family member with an established diagnosis of memory loss, MCI, or any cause of dementia: the caregiver needed to have a family member with MCI or early ADRD who frequently spent at least 4-hour stretches alone at home apart from any caregiver in the last 12 months; and (3) male or female clinician with experience in managing patients with MCI or ADRD: clinician participants must have provided care to patients with cognitive impairment and/or supported families of such patients for at least one year.

### Recruitment

Our goal was to recruit at least six participants from each of the 3 key stakeholder groups: people with MCI or ADRD, caregivers, and clinicians. To recruit people with dementia, we identified patients with MCI or early ADRD from the current VA Bedford outpatient primary care and dementia clinics using electronic medical records. Once identified, the VA Bedford study coordinator (JM) reviewed the electronic medical records of the patients to determine whether they met the inclusion criteria. Those who met the inclusion criteria were mailed an invitation letter for participation in the study; the letter included a phone number to call should they wish to opt out of the study. The study coordinator made a recruitment phone call to patients who did not call back to opt out.

Family caregivers were recruited through family relationships with people with dementia who were recruited for this study or through inputs from VA clinical providers. Caregivers also received a letter of invitation, and the study coordinator followed up with telephone calls or email.

VA Bedford providers with expertise and experience in managing patients with MCI or ADRD were recruited by the VA study principal investigator (LM) and/or study coordinator via email. Using an opt-in approach, the recruitment email instructed the invited clinician to contact the study coordinator if they were willing to participate. Up to 3 emails were sent to each clinician.

### Interview Guide

The interview guide contained semistructured questions to elicit open responses from participants, as well as structured questions that asked participants to rate their responses on a Likert scale as well as to categorize options (eg, from most useful to least useful) and then provide a rationale for their response. Questions related to perceived usefulness and perceived ease of use were used to assist in understanding stakeholders’ preferences regarding technology and identify adaptations that can be made to the robot (ie, the mobile robot, as both concepts may affect stakeholders’ attitude toward using the mobile robot, which can then influence their behavioral intention to use the mobile robot and thereafter, affect actual use). We conducted a literature review to understand the types of questions and qualitative themes found in previous research studies relating to mobile telepresence robots as well as other types of robots that have been developed to assist people with ADRD [18-23]. On the basis of this literature review, we developed interview questions that focused on domains that we established a priori to help guide our understanding of how stakeholders perceive the mobile robot’s usefulness and ease of use: (1) technology use, (2) design and functionality of the robot, (3) future applications to be incorporated into the robot, and (4) overall opinions about the robot. We established these as key domains because they would allow us to elicit information from stakeholders to accomplish this study’s overall purpose and specific aims. We asked participants similar questions across all stakeholder groups. Table 1 provides relevant questions for these domains. Of note, for the question “Please let me know how you would rank these applications that could be used or built into the robot (from most useful to least useful),” the 6 categories of applications were chosen by subject-matter expert team members with extensive clinical knowledge (LM) and technical knowledge (AR)—based on their professional experience in working with people with MCI or ADRD, conducting research on robots, and/or developing robots. The subject-matter experts met to brainstorm and discuss what types of applications would enhance the mobile telepresence robot for this population and chose the 6 applications that would be the most feasible to add to the robot.

**Table 1:** Domains and relevant interview questions.

Domain	Interview question
Technology use	How often do you use the internet? How often do you use the internet for health-related reasons or to get answers to health-related questions? What kinds of technology do you regularly use at home? Thinking about the technology you just mentioned you regularly use: choose one of the following that best describes your comfort level. I am very comfortable using this technology. I am somewhat comfortable using this technology. I am somewhat uncomfortable using this technology. I am very uncomfortable using this technology. Not sure. Do you use any applications for health purposes?
Design and functionality of the robot	What do you like about the design and functionality of the robot? What do you not like about the design and functionality of the robot? Do you have any suggestions for changes to be made to the design and functionality of the robot?
Future applications to be incorporated into the robot	What kinds of new applications would be useful to build into the robot? Please let me know how you would rank these applications that could be used/built into the robot (from most useful to least useful). Medication reminders or dispensing Communication with family/caregivers Reminders about the day’s schedule Communication with medical staff/providers Emergency help access Social stimulation activities such as games or reading the news
Overall opinions about the robot	On the basis of the video/materials we showed you and what you now know about the current version of the robot, what is your overall opinion about the robot (1=useless to 10=excellent)? Why did you give that rating? What is the greatest value you think it would provide? Do you foresee any challenges in using the robot?

### Data Collection

From July to August 2019, a total of 3 health service researchers (MS, JM, and JS) who are experienced in qualitative methods, conducted the semistructured interviews. Each stakeholder participated in 1 in-person individual or group interview, which lasted approximately 60 minutes. Interviews were conducted either at the VA Bedford main facility or at the participant’s home. People with MCI or ADRD and family caregiver participants received a US $50 gift card for their participation in the interview and an additional US $30 gift card to compensate for travel time if they participated in person at VA Bedford. To thank them for their time and insight, the VA clinician participants received a modest meal, which did not exceed a value of US $20 per person. Before the interviews, all participants provided informed consent and agreed to be audio-recorded for transcription purposes.

**Figure 1:**
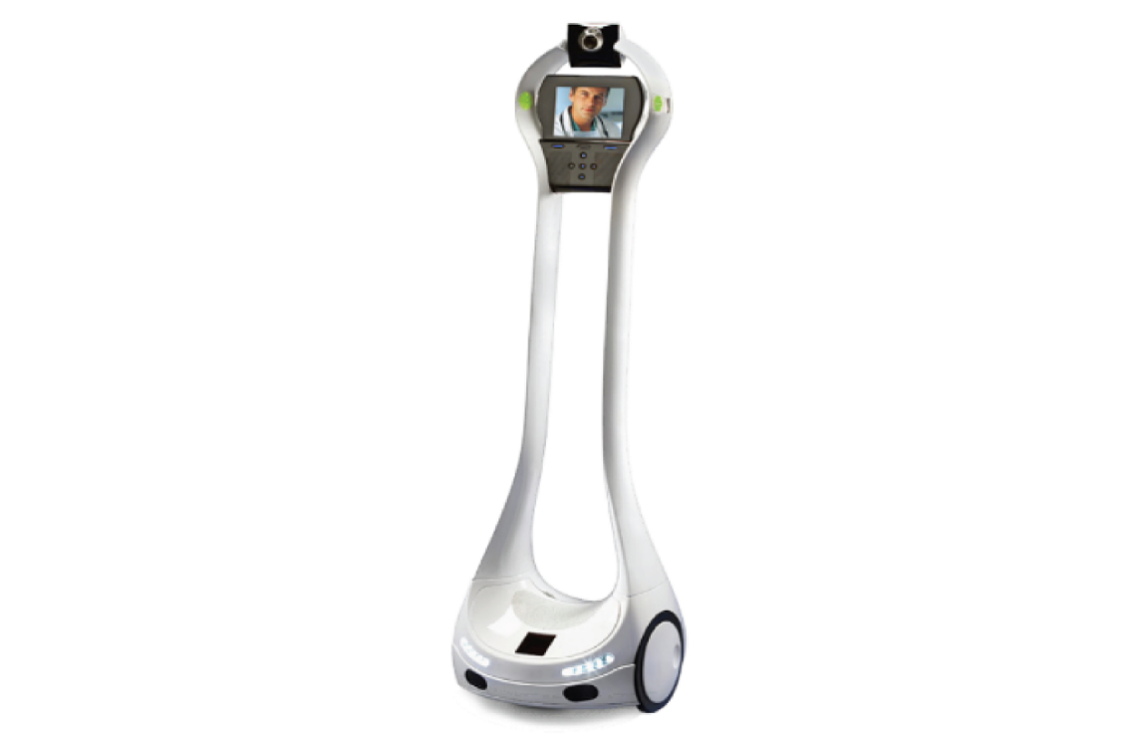
Mobile telepresence robot used in the study.

After obtaining consent, the interviews began by asking participants to complete a stakeholder group–specific demographics questionnaire. As part of the interview process, we also provided participants with information about the existing, commercially available mobile telepresence robot, which included a photo (Figure 1) and a brief explanation.

This robot is a wheeled, upright (approximately 122 cm tall), self-propelled device that weighs 8.6 kilograms with a 14.5-cm screen; it moves about under remote control and provides 2-way video communication between a remote user who pilots the robot through an app accessed via a computer, tablet, or smartphone and a person who is physically present with the robot. In addition, we presented a video (television news segment) to visually display the robot’s current capabilities. The video featured a student who was immunocompromised and could not attend school in person. However, the student was able to attend school and remain in the classroom virtually through the mobile telepresence robot. Through this video, the participants were able to see how the robot displayed the student’s face on the screen and enabled her to talk to and socialize with her classmates and teacher in the classroom and how she could control the robot, allowing it to move through the classroom and down the hallways. We then asked the participants questions from the interview guide.

### Data Analysis

Interview transcripts were the primary data source for the directed content analysis. Coding and data analysis focused on four a priori domains: (1) technology use, (2) design and functionality of the robot, (3) future applications to be incorporated into the robot, and (4) overall opinions about the robot. Using a code book that contained domains with definitions, the core analytic team (MS and JS) jointly coded 1 interview transcript and examined the transcript for evidence of the established domains. The 2 researchers discussed the coding process, resolved any discrepancies, and reached a consensus on definitions and coding classification. Thereafter, the transcripts were divided and coded separately by the 2 researchers using NVivo (version 12; QSR International) qualitative software.

Upon completion of coding, we generated coding reports for each domain and stakeholder group. The data in the coding reports were reviewed and then synthesized into a matrix, which allowed us to organize the data to facilitate the final interpretation. Next, we used a matrix analysis technique to assess patterns within and across stakeholder groups [24,25]. Thereafter, for each of the 4 domains, we identified salient themes while noting similarities and variations across stakeholder groups. In addition, we compared patterns in the Likert ratings and responses across stakeholder groups. To ensure wider perspectives, we included study team members (JM, LM, and AR) beyond the core analytic team to review and comment on the study findings.

## Results

### Participant Characteristics

Overall, a majority of participants (20/21, 95%) were White across all 3 stakeholder groups. The 6 people with MCI or ADRD in our study sample were men (mean age 73 years, range 69-80 years), with 4 (67%) of these participants living with a family caregiver and 2 (33%) were living alone. All 9 caregiver participants in this study were women (mean age 60 years, range 42-80 years). Of the 9 participants, caregivers’ relationships with family members with MCI or ADRD included 6 (67%) participants who were the adult child or child-in-law of the family member with MCI or ADRD and 3 (33%) participants who were the spouse or partner. Of the 6 clinician participants, 4 (67%) were women and 2 (33%) were men. All had advanced degrees ranging from master’s to doctoral, with an average of 20 years of experience in their respective fields. They estimated that, on average, 89% of their patients were aged ≥60 years, and 76% of their patients had cognitive impairment.

### Person With Dementia and Family Caregiver Technology Use

Although most individuals with MCI or ADRD reported that they were able to use their mobile phones for basic functions (eg, phone calls or SMS text messages), overall, they reported lower use of technology and the internet than caregivers, who reported higher use:

I don’t really use [the internet] that much. I’m not interested and it’s confusing.

Person A with MCI or ADRD

If I walk out the door without my phone, no I go back in the house to get it. It’s not one of these, oh yeah I forgot it I’ll get it when I get home.

Family caregiver A

Most individuals with MCI or ADRD had never used the internet or applications for health-related reasons or to get answers to health-related questions; similarly, caregivers also reported lower use for health-related reasons or questions. However, most caregivers reported using at least one app for health purposes.

### Design and Functionality of the Robot

Overall, stakeholders across all 3 groups expressed that they liked the mobility and size of the robot (approximately 122 cm tall), as well as the ability to connect and communicate in an interactive way. However, they expressed concerns related to the size of the robot for individuals living in smaller apartments and trailers, as well as the stability of the robot, questioning whether it could tip over easily. In addition, stakeholders queried whether the screen size and quality (14.5 cm and 320×240-pixel display) would provide enough support for those with visual impairments who would need a screen with higher-quality contrast and that could display larger text or image. Across all stakeholder groups, voice commands and 1-touch lifesaving or help buttons were the 2 top suggestions offered:

I would [add voice activation] especially if someone has physical trouble, like if they’re [a person with disability]. That would be a big help.

Person B with MCI or ADRD

I don’t know whether my dad would find it as easy to use unless it is voice activated. Now [that] he has a voice control for the television [he] loves it.

Family caregiver B

[I]f there’s a trigger, [a person] just falls, and somebody is on the floor and there’s some device that [the robot] has that alerts you whether it be the bracelet or something. [A person] can activate an emergency system and then as opposed to having a false alarm, the person, the communicator or the emergency system would be able to say, oh, Mr. Smith, you’re on the floor. It would help with that. And that would actually be extremely important.

Clinician A

As presented in Table 2, stakeholders offered several suggestions on what features could be added to the robot and provided input on how those features could help improve the design, functionality, and use of the robot in the context of people with MCI or ADRD. The features listed in Table 2 are presented from most often suggested to least often suggested across all stakeholder groups. Adding a voice command feature to the robot was suggested most often, whereas adding lights to the robot was the least often suggested feature.

**Table 2:** Feature to add to the robot, number of stakeholders who requested each feature, and stakeholders’ feedback.

Feature to add to the robot	Stakeholders who requested each feature (N=21),^a^ n (%)	Stakeholders’ feedback on how this can help to improve the robot’s design and functionality or help stakeholders
Voice command; option to change the voice (eg, male vs female voice)	Person with MCI^b^ or ADRD^c^: 5 (83) Caregiver: 4 (44) Clinician: 6 (100)	Helps with safety (eg, more easily able to call for emergency help as well as call family members and providers) Helps with feeling comfortable with robot (choice of male voice vs female voice)
Screen adjustment capacity (eg, photograph and touch screen zoom capability)	Person with MCI or ADRD: 0 (0) Caregiver: 5 (56) Clinician: 5 (83)	Helps in emergency situations, during telehealth appointments Helps those who have visual impairments or when visual adjustments are needed
Size, collapsible, or foldable options	Person with MCI or ADRD: 3 (50) Caregiver: 3 (33) Clinician: 3 (50)	Helps those who live in smaller dwellings Helps with ease of moving the robot, if carrying it from room to room or to another floor of the house
Buttons (eg, lifesaving or help call, or on or off)	Person with MCI or ADRD: 2 (33) Caregiver: 2 (22) Clinician: 4 (67)	Helps with safety
Volume adjustments	Person with MCI or ADRD: 0 (0) Caregiver: 2 (22) Clinician: 5 (83)	Helps those with hearing impairments
Alarm, bell, or beeping sound	Person with MCI or ADRD: 0 (0) Caregiver: 3 (33) Clinician: 3 (50)	Helps to alert when someone is calling/dialing in Helps to alert a person that robot is near them so the person is not startled
Customizable color, print, or pattern options	Person with MCI or ADRD: 2 (0) Caregiver: 3 (33) Clinician: 1 (17)	Helps with connection and comfort with the robot (eg, select a color or pattern/print that the patients like)
Attachments (eg, arms, handles, or cupholders)	Person with MCI or ADRD: 0 (0) Caregiver: 3 (33) Clinician: 3 (50)	Helps patients, particularly with mobility challenges, around the house Helps caregivers to have more control in the home virtually (eg, use the robot to pick up and look at medication bottles, start the microwave, or pick up clutter) Helps the robot go upstairs in a lift
Entertainment options (eg, music, television, or movies)	Person with MCI or ADRD: 0 (0) Caregiver: 2 (22) Clinician: 3 (50)	Helps people feel more engaged and comforted by something familiar and enjoyable to them Helps to increase participation
Lights	Person with MCI or ADRD: 0 (0) Caregiver: 1 (11) Clinician: 1 (17)	Helps those who have visual impairments Helps with nighttime vision for the robot to be able to gather visual information in a dimly lit house Helps with lights around the robot to see the robot easily if the lights are off or the house is dimly lit

^a^Person with mild cognitive impairment or Alzheimer disease and related dementias: n=6; caregiver: n=9; clinician: n=6.

^b^MCI: mild cognitive impairment.

^c^ADRD: Alzheimer disease and related dementias.

### Future Applications to Incorporate Into the Robot

#### Overview

**Figure 2:**
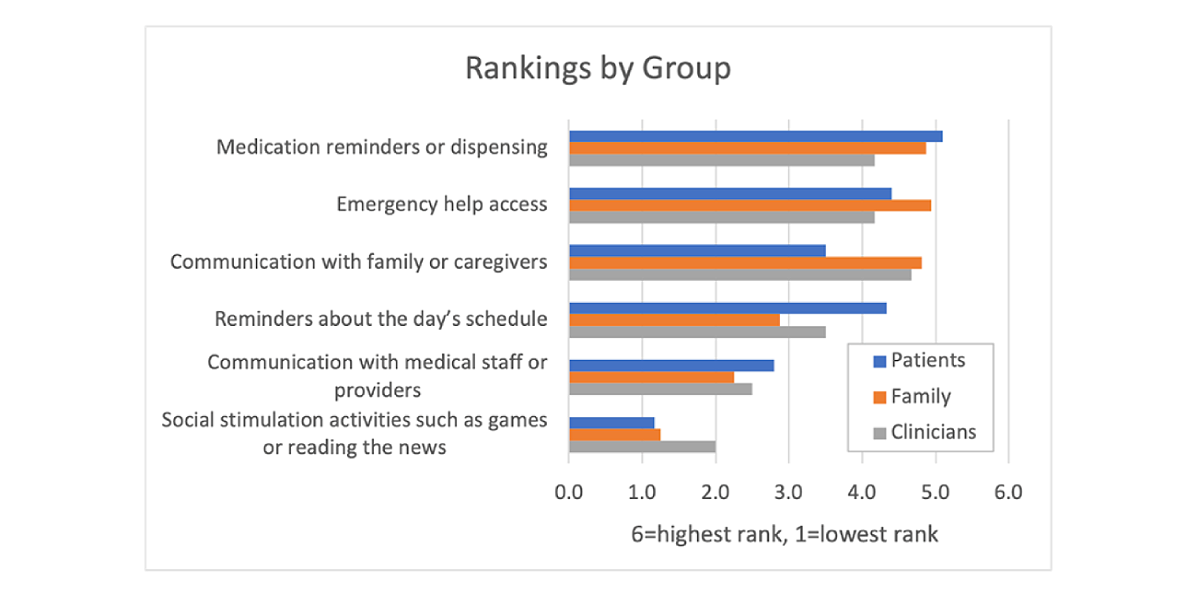
Mean score of the 6 applications ranked across stakeholder groups.

When stakeholders in each of the groups were asked by the study team to rank 6 possible robot applications (ie, medication reminders or dispensing, communication with family or caregivers, reminders about the day’s schedule, communication with medical staff or providers, emergency help access, and social stimulation activities such as games or reading the news) from most useful to least useful, overall across stakeholder groups, participants rated *medication reminders or dispensing* and *emergency help access* as the 2 most useful applications. For people with MCI or ADRD, the top 3 applications were *medication reminders or dispensing*, *emergency help access*, and *reminders about the day’s schedule*. Similarly, caregivers and clinicians included *emergency help access* and *medication reminders or dispensing* in their top 3 rankings. However, although *communication with family/caregivers* ranked the highest and second highest choice among clinicians and caregivers, respectively, among people with MCI or ADRD, this application ranked fourth overall. Across all 3 stakeholder groups, *social stimulation activities such as games or reading news* ranked the lowest. Figure 2 presents the mean scores of the 6 applications ranked across each stakeholder group with 1 being the lowest and 6 being the highest.

In addition to ranking the options of possible applications provided by the study team, stakeholders across the 3 groups offered their own specific suggestions on the types of applications they perceived as useful to include in the mobile robot to enhance the opportunity for individuals with MCI or ADRD to live at home independently. We categorized these suggestions across the 3 stakeholder groups into 4 types of applications: health-related alerts, reminder prompts, smart-home–related applications, and social, entertainment, or well-being applications.

#### Health-Related Alerts

Suggested applications include health-related alerts such as those that could inform providers of a change in patients’ blood sugar or blood pressure levels, as well as those that could be triggered if a person falls. Similar to a bracelet or necklace alert, activation and connection to a previously imputed emergency response system such as family members, ambulance, police, emergency medical technicians, and/or health care providers, could occur.

#### Reminders

Suggested applications include those offering real-time, step-by-step prompts on how to complete multistep activities such as getting dressed, making a microwave meal, or turning the oven on/off; those about medication, doctors’ appointments, other scheduled activities, and wake-up or bedtime prompts, especially for those who have sleep problems; and those that can prompt a person with MCI or ADRD to perform and help with activities of daily living or instrumental activities of daily living (eg, prompt for eating, bathing, or bathroom use).

#### Smart-Home–Related Applications

Suggested applications include smart-home technology integration, in which the robot can detect motion, connect to a home security alert, and recognize home hazards (eg, smoke and carbon monoxide detection, or tripping hazards). In addition, the robot can work similarly to or in conjunction with a voice-activated smart-home device (eg, the robot turns on or off appliances in the home).

#### Social, Entertainment, or Well-being Applications

Suggested applications that could enhance the well-being and comfort of a person with MCI or ADRD include community or social connection applications, such as ride share applications that could assist transportation arrangement and allow individuals with MCI or ADRD to go out into the community independently (eg, attending religious groups for social connection); those that stream music, movies, and television shows either directly on the robot’s screen or projected onto a wall, which could also be turned on virtually by a caregiver; and games to help with cognitive engagement and exercises to help with balance and mobility. In addition, an app related to identifying family members or friends to assist with recall could enhance social connection and well-being, such as integrating contact numbers of specific family members or friends into the robot, uploading photos and voice recordings of their family members or friends, and inputting specific memories with family members or friends and important dates (eg, birthdays and anniversaries) into the robot.

### Overall Opinions About the Robot

Of the 3 stakeholder groups, caregivers reported the highest overall rating for the robot (mean 8.1; rated on a scale of 1 to 10). All caregivers (9/9, 100%) positively perceived the robot and liked that they could virtually check in and communicate with their family members, especially if their family members were to be left alone for an extended period. A caregiver described how her family member may trust the robot more than humans to help around the house:

If I can control [the robot] from afar that's helpful. If it can do some things on its own like little reminders and things, that's helpful. It just serves as a bridge and the fact -- like I said for my dad where he doesn't trust humans to come into the house maybe he would let a machine.

Family caregiver C

However, of the 9 caregiver stakeholders, 7 (78%) noted that additional features and applications would have to be added to the robot for it to be more useful; thus, the reason for not rating the robot a 10.

Overall, ratings from the clinician and people with MCI or ADRD stakeholder groups (mean 7.4 and 6.4, respectively) were slightly lower than the caregiver group, noting that additional features and applications had to be added to the robot for it to be more useful. Similar to the caregiver group, all clinicians (6/6, 100%) expressed positive perceptions about the robot and its potential to increase a person’s independence and ability to stay home alone:

What we worry about with people at that stage is that they can continue to do as much as they can, but they need support. And we do know more and more people are living alone. And often the only option is bringing in a home health aide who again might not be the person they want to see there. Or they end up needing to move often. I just think that this will provide them the support that they need, if they welcome it, to be able to keep doing what they’re doing as long as possible. It seems like it’s enough support. But it’s not too much. Especially if it’s like a menu of things. If it’s like they don’t have to accept all of these things at once. You could titrate [support].

Clinician B

However, one of the main reasons clinicians rated the robot lower on the scale was that they perceived that the robot in its current state did not offer much beyond what could be done with other virtual connection platforms such as Skype, Facetime, or other modalities used in video telehealth sessions:

I think that a lot of the things that it’s used for can already be done through Facetime or Skype or what not. I think that there’s a little bit extra better quality to this.

Clinician C

Although the robot was perceived to be beneficial in terms of living independently if additional features and applications were added, 50% (3/6) of the people with MCI or ADRD rated the robot lower on the scale for two main reasons: (1) they perceived that they did not need the robot as they had family members who cared for them and (2) they did not know if they could use the robot in their homes because of challenges with size and mobility:

We don’t have [the robot] now. We do fine without [the robot]. I got my wife. I call her my guidance counselor...I wouldn’t use it...trying to figure out how to use [the robot would be a challenge].

Person C with MCI or ADRD

If [my children] lived really far away, I could see where that might be nice to see them once in a while and be able to talk to them instead of just on the phone. It would be good that way, but [my] children don’t live far away so me personally it’s not exciting to have.

Person D with MCI or ADRD

[F]rom what you say [the robot] can do I’d be happy with it but it’s going to get in the way. It’s going to have to use the other bedroom all the time...[The robot is] too tall and too wide.

Person E with MCI or ADRD

In addition, 1 person with MCI or ADRD, who noted that he did not really have any family or friends, reported that the robot could provide companionship to someone who lives alone:

I think [the robot] would offer companionship because like I said I don’t have any family really. I don’t have any friends at all so I’m kind of alone all the time. It doesn’t really bother me. I get used to it after a while. But yeah, I think for somebody like me, [the robot] would offer companionship.

Person F with MCI or ADRD

### Greatest Value

All (6/6, 100%) stakeholders with MCI or ADRD reported that the greatest value provided by the robot would be help in case of emergency and/or fewer in-person visits to the doctor’s office (ie, ability to conduct tele-visits):

[O]n an average I’ll be honest with you I avoid the doctor as much as possible. I avoid them like the plague because it’s something I don’t like—the smell of hospitals. They make me sad and they’re a depressing place. That’s another reason I don’t like going...[I]f I can avoid going to the doctor and I can just sit at home and say [to the doctor] see this, [the robot would help].

Person F with MCI or ADRD

[I]f there was a button that [people] could just push on the [robot] itself. [The robot] would have programmed in your address and how to get into the house for the emergency responders. And if it could automatically open a door, if the doors are locked.

Person B with MCI or ADRD

Similar to people with MCI or ADRD, all the clinician stakeholders (6/6, 100%) agreed that the greatest value of the robot would be its ability to provide help in case of an emergency, as well as help their patients with safety at home and increase a person’s independence, particularly if additional design features and applications were incorporated:

[T]he security feature. So often we hear someone falls or something very minor escalates, like they leave a potholder on the stove and turn and walk away and the potholder burns and there’s a kitchen fire. And it’s not even necessarily the gravity of the act. I think that it’s the fear of what could happen ends up curtailing the person’s independence...[T]he daughter or son being able to say [using the robot], what’s that box doing in the middle of the floor? Move that to the right. [This] could prevent a fall.

Clinician B

Clinicians also highlighted that the robot offers an alternative for in-person doctor visits and can provide clinicians with access to the patient during an emergency. Although caregivers noted similar opinions regarding emergency help, some discussed that in-person doctor’s appointments helped their family members get out of the house and increased social interactions.

All caregivers (9/9, 100%) reported that remote video communication with their family members with MCI or ADRD was one of the greatest values of the robot; this technology allows caregivers to perform quick check-ins or longer calls with their family members, as well as observe what is happening when caregivers are unable to make an in-person visit:

Being able to look at the house to see by chance maybe there’s something on the floor that they might trip over that you could say [to the robot] go such and such a place, there’s something on the floor, pick it up. Being able to [see that] without actually physically being there. I live close to my parents, but some people [live] hours away. Having something like this would probably help with a lot of people [with ADRD] being able to stay in their own homes.

Family Caregiver A

Really to be able to see her all the time [is the greatest value]. To see what she’s doing because I’ve gone to the house and the stove is on. I’m like why is the stove on? I don’t know. I’m like okay we need to be able to see her all the time.

Family Caregiver D

### Concerns

Although there were many positive responses to the mobile telepresence robot, all 3 stakeholder groups expressed concerns about using the robot, including technology usability, privacy, and security. In all, 50% (3/6) of people with MCI or ADRD were concerned about the technology needed to operate the robot because they did not own a computer/smartphone and lacked knowledge of how to use this technology:

I think if [the robot] was voice commanded so you could speak to it to do things that would be the biggest help. But otherwise, whoever needs it has to make sure they either have a phone that they know how to use, [or] can use it [like] a laptop...I think people who might be in need of that are probably going to be older and not as likely to be tech savvy and be able to use computers and phones.

Person D with MCI or ADRD

In all, 44% (4/9) of the caregivers and 67% (4/6) of the clinicians also expressed similar concerns about the ability to use the robot by people with MCI or ADRD (eg, ability to fix the robot if presented with technological challenges, lack of Wi-Fi, or equipment needed to operate the robot):

I think my mother would be good with [the robot]. I think my father, it might be challenging for him only because I don’t feel that he has the attention span. I think that maybe if this machine was in front of him and he saw what it could do, I think it might spark a little interest.

Family Caregiver E

I think [there are a lot of] unknowns. We only got to see the interface if somebody was talking on the other end. I think there are a lot of other things that are sort of unclear including how it would work, how it would sync with other devices, that sort of thing. Ease of use.

Clinician D

To address these concerns, people with MCI or ADRD and caregivers suggested the development of a step-by-step tutorial (video and written instructions) that could teach users how to operate the robot. In contrast, a clinician perceived that people with MCI or ADRD would not experience technological challenges in operating the robot:

You’d be surprised that patients are starting to become more tech savvy. Believe it—even cognitively impaired people. I’ve got a couple on my video conferencing, they’re doing okay, and the family members are also [okay] because everybody [uses] Skype so they’re starting to be more at ease with it.

Clinician A

Of the 6 people, although 1 (17%) with MCI or ADRD was concerned about privacy or security, the other 5 (83%) were not. However, caregivers and clinicians expressed more concerns about privacy and security. They discussed the tension between caregivers wanting to check in with their family members but feeling as though they may be infringing on their family members’ privacy when controlling the robot. Concerns regarding privacy and patient dignity (eg, when a patient is changing or going to the bathroom), especially when there are multiple permissible remote users, were also noted:

[F]or the most part, he would appreciate it although there are probably moments where it may feel intrusive...[need to make the robot] so it’s not intrusive into any person’s private moments...I think that’s the only thing I would think about if someone’s like, Hi, daughter, didn’t realize you were right here.

Family Caregiver F

I’m trying to picture a sort of dual control of the on/off [which would help with] the privacy concern. I mean the problem is that it helps him with the privacy concern but it doesn’t help me with the check on him. Cause he is liable to turn it off and forget that he turned it off and then if I can turn it on anytime I need to or want to—how does that give him the privacy that he is looking for. So there’s that tension there.

Family Caregiver B

[T]here could be privacy issues. All of that needs to be [thought about] to avoid abandonment, meaning the [robot] gets driven into a corner, thrown down the stairs...I could see, especially if someone’s losing their cognition. How do we make sure that we embed this in a way that’s the person’s needs are respected and that it’s done on their terms? As much as the family and the clinician’s terms. How do we remind them, for example, if they have a [tele-] appointment with their doctor how do we make sure that we give them plenty of reminders so that a face just doesn’t show up in the middle of them watching [TV] in their underwear? And all of the sudden they’re embarrassed and they say I never want to do this again. How do we build in enough fail safe features for privacy?

Clinician B

To help alleviate concerns, caregiver and clinician stakeholders noted that privacy and security terms would need to be spelled out clearly, such as those related to who would have main control over the robot, if any data are recorded and the security associated with it, and whether someone could easily access the robot and obtain private information.

## Discussion

### Principal Findings

We conducted this qualitative study to obtain feedback from the 3 groups of key stakeholders (people with MCI or ADRD, family caregivers, and clinicians) on how to adapt an existing, commercially available mobile telepresence robot to specifically support individuals with MCI or early ADRD so that they can continue to live at home independently. Through these interviews, we received consistent feedback across groups, which could enhance the robot’s usability. Suggestions that differed by stakeholder group provided us with a complete understanding of the adaptations that need to be made to strengthen the utility of the robot for all stakeholder groups.

People with MCI or ADRD, family caregivers, and clinicians all described multiple possible updates to the design and functionality as well as the applications of the remotely navigable telepresence robot. Family caregivers and clinicians clearly perceived the need for additional support for people with MCI or early stage ADRD to allow them to live alone or be left alone for long periods. Both stakeholder groups felt that the augmented version of the mobile telepresence robot in our study could play an important role. Compared with clinicians and family caregivers who were the most enthusiastic of the 3 stakeholder groups, people with MCI or ADRD reported lower ratings for the robot. Despite these lower ratings from people with MCI or ADRD, many stakeholders across all groups perceived that the robot had the potential to increase a person’s independence and ability to stay at home. For example, stakeholders perceived the videoconferencing function (already available in the robot) to be useful in facilitating communication with friends or family members and for video telehealth visits with providers, which helps strengthen relationships by bridging the distance between individuals [26]. However, similar to previous research that highlights the importance of developing robots based on stakeholder feedback [18,19,27], incorporating several adaptations regarding the robot’s design, functionality, and applications would be critical to enhance use for their needs, such as additional development of voice command and help button functions as well as applications related to medication reminders or dispensers and emergency response access [20]. These were perceived as critical features or applications to help people with MCI or ADRD maintain independent living at home; stakeholders raised concerns that these may be barriers to adoption if not incorporated. Among the 3 stakeholder groups, medication reminders and emergency help access ranked the highest, with reminders regarding daily schedules, rounding out the top 3 for those with MCI or ADRD. Although all 3 functions were beyond what the robot could do at the time, the fact that 2 of the 3 related specifically to help with memory decline highlights the importance of engaging disease-specific stakeholders in such studies. Similarly, concerns about the ease of use of the technology with strong recommendations from all 3 groups regarding the integration of voice commands also underline the benefits of including people with MCI or ADRD in the development of products or interventions for which they are the primary target as well as family caregivers and clinicians. Our findings echo previous studies that highlight the importance of aligning and customizing technology functions and applications to end-product users [12,18,19,28-31].

Our results underscore the importance of engaging and obtaining end-user input from different groups of stakeholders in technology development—the individual with MCI or ADRD, the caregiver, and the clinician—which provides for the opportunity to tailor according to the needs and interests of all who are involved in the care of people with MCI or ADRD. Although this is an important component of technology development, to the best of our knowledge, only a few previous studies have interviewed all 3 groups [12,18]. In addition, engaging community-dwelling people with MCI or ADRD is a critical and feasible component of technology development; however, studies have usually lacked the involvement of people with MCI or ADRD in technology development [16]. This lack of involvement may lead to the implementation of technology that is not tailored or suitable to the individuals who the technology intends to serve [16]. Family caregivers are also important stakeholders and are quite likely one of the most critical given how this type of technology (mobile telepresence robots) may help alleviate caregiver burden. Caregivers of people with MCI or ADRD are their primary advocates; the primary people with whom they communicate with; and may usually be the primary decision-makers in the household. By involving caregivers as a stakeholder group in our study, we were able to obtain input on what types of features and applications of the mobile telepresence robot can help reduce caregiver burden.

The feedback provided on the adaptations to the mobile telepresence robot and possible applications for the inclusion of all 3 stakeholder groups aligned with concepts such as perceived ease of use and usefulness as well as trust [32]. These concepts are critical to the adaptation of mobile telepresence robots in health care communication settings and can lead to barriers in adoption if not resolved. Previous studies have noted that barriers to the acceptance of mobile telepresence robots included challenges in using technology and concerns about the ability of older adults to operate the robots [18,33,34]. In our study, compared with caregivers, people with MCI or ADRD reported overall lower use of technology and the internet, which may have affected their perceptions about the usefulness and ease of use of mobile telepresence robots. People with MCI or ADRD and caregivers expressed concerns regarding the technology use required by people with MCI or ADRD to operate the robot. One of the most often suggested design features that stakeholders across the 3 groups wanted (particularly people with MCI or ADRD) to have incorporated into the robot was voice command technology. This simple design improvement has the potential to increase the perceived ease of use and usefulness of robots. In addition, stakeholder feedback can assist in understanding preferences that may potentially enhance trust, thereby leading to a higher adoption of technology. Similar to previous research [19], people with MCI or ADRD in our study did not seem to be concerned with privacy and security. However, in contrast to previous research on caregivers who were more likely to perceive no ethical dilemma when balancing this with the safety of people with dementia [35], privacy and security were the main concerns expressed by both caregivers and clinicians. Caregivers and clinicians were unable to offer solutions that they felt would alleviate their concerns but noted that these challenges would need to be resolved for end users, such as themselves, to trust the robot. Given the dearth of studies focusing on privacy and security with robots for people with MCI or ADRD, additional exploration is warranted and should be incorporated into future research [13].

Although applications related to social stimulation activities (eg, *games or reading the news*) ranked lowest across all 3 stakeholder groups, stakeholders offered suggestions in terms of what types of social, entertainment, or well-being applications they would want to see in the robot; for example, ride share applications that could assist transportation arrangements and allow individuals with MCI or ADRD to go out into the community independently and applications that could assist with recall to help identify family members or friends as well as specific memories and important dates (eg, birthdays and anniversaries). These suggested applications to enhance the robot are important because they may enable people with MCI or ADRD to be even more socially engaged and connected to their family and friends—a key component of what a telepresence robot is supposed to be doing. In particular, as highlighted during the COVID-19 pandemic, social interactions and connections are critical. The pandemic heightened feelings of loneliness as the number of older adults who were socially isolated grew and they were unable to participate in social activities, thus significantly affecting their mental health [36]. Before the COVID-19 pandemic, about one-fourth of the older adults living in the community in the United States were thought to be socially isolated, with approximately 40% of these older adults reporting feeling lonely [37]. Compared with well-established risk factors such as smoking, high blood pressure, and obesity [37-39], social isolation and loneliness can increase the risk of depression, poorer cognitive function, and dementia leading to an increased risk of mortality and morbidity [39]. In the United States, deaths caused by Alzheimer disease and dementia have increased by 16% during the COVID-19 pandemic [1]. The pandemic has shown the importance of developing innovative technology that can improve social connections and support for older adults [36,40]. Our study results echo findings from another study of community-dwelling older adults, highlighting that one of the roles of a mobile robot was to provide friendship or companionship or to provide help [27]. In addition, our study also underscores stakeholders’ perceptions that one of the greatest values of a mobile telepresence robot is the connection that it can offer to people with MCI or ADRD through remote video communication with family members and friends. Although the robot, as is, provides access and social connections, integrating stakeholders’ suggestions on applications into the robot would only further enhance engagement and social connections.

Through this qualitative study, we were able to obtain feedback from key stakeholders and identify the types of features and adaptations they prefer on an existing, commercially available mobile telepresence robot to enhance the support of at-home care for people with MCI or mild to moderate ADRD. As discussed earlier, stakeholders offer several suggestions regarding their desires. Some of these features and adaptations may be more feasible than others. For example, participants noted voice command as a highly desired feature, which is becoming more common in robots (eg, Alexa and Siri). A technology company that focuses on designing and engineering robots may find this feature more feasible to include in a mobile telepresence robot because of the advancements in this technology compared with other desired features such as attachments (eg, arms, handles, or cupholders), which may be challenging because of the robot’s center of gravity, or a robot that cleans the house, which is a function that is beyond current capabilities. Moving forward, an important next step for companies that design and engineer robots is to assess the feasibility of the desires from an engineering perspective and balance the challenges in fulfilling the desired features and functions of the stakeholders while also ensuring the robot’s usability for the target population.

### Limitations

There are a few limitations to our study that should be acknowledged. Similar to the challenges faced by most qualitative studies, participants volunteered to be a part of the study, and interviews elicited those particular perspectives; thus, interviews may have been subject to selection bias. In addition, stakeholders only viewed the robot through a video and did not see the robot in person; this may have limited their ability to visualize and understand the robot’s full capacity of what it could offer. Our study participants were all from the Greater Boston area; thus, we only had a sample of participants from 1 geographic area. In addition, because of the small sample size, particularly within each stakeholder group, we were unable to compare similarities and differences in feedback within each stakeholder group, such as whether caregivers of varying education levels made similar or different suggestions on features and applications to incorporate into a mobile telepresence robot. Because this was a qualitative research study, we did not collect large quantitative data sets that would allow comparisons with the general or larger Veteran patient population. These comparisons are interesting areas for future research. However, a major strength of our work is that, to our knowledge, this is one of the few studies to elicit stakeholder feedback about adaptations that can be made to a telepresence robot from all 3 user groups: people with MCI or ADRD, family caregivers, and clinicians. In addition, our findings are generalizable to other assisted technologies for individuals with MCI or ADRD, caregivers, and clinicians.

### Conclusions

Recognizing the central role of each of these 3 end-user groups (people with MCI or ADRD, caregivers, and clinicians) is crucial for the development and adoption of technology for people with MCI or ADRD to help them remain living in the community. We learned from these 3 stakeholder groups what a mobile telepresence robot can and cannot do for people with MCI or ADRD; a robot may help to increase social connection and reduce feelings of loneliness, increase medication compliance and adherence to health routines, increase the independence of people with MCI or ADRD, and increase caregiver well-being [41]. Our results provide insights into the ways in which a mobile telepresence robot can be adapted to enhance utility from the perspective of all 3 stakeholder groups, which can ultimately be used to develop autonomous robotics features. Future research should continue to incorporate the perspectives of all 3 stakeholder groups in studies to further investigate what adaptations are needed for different types of robots to ensure optimal use by all end users.

## Abbreviations

ADRD: Alzheimer disease and related dementias
MCI: mild cognitive impairment
VA: Veterans Affairs

## References

[1] (2021). Alzheimer's disease facts and figures. Alzheimer's Association.

[2] (2019). Alzheimer's Disease and Healthy Aging: Caregiving for a person with Alzheimer's disease or a related dementia. Centers for Disease Control and Prevention.

[3] (2020). Alzheimer's disease caregivers: fact sheet. Alzheimer's Association.

[4] Moyle W, Arnautovska U, Ownsworth T, Jones C (2017). Potential of telepresence robots to enhance social connectedness in older adults with dementia: an integrative review of feasibility. Int Psychogeriatr.

[5] Broekens J, Heerink M, Rosendal H (2009). Assistive social robots in elderly care: a review. Gerontechnology.

[6] Kruse CS, Fohn J, Umunnakwe G, Patel K, Patel S (2020). Evaluating the facilitators, barriers, and medical outcomes commensurate with the use of assistive technology to support people with dementia: a systematic review literature. Healthcare (Basel).

[7] Kachouie R, Sedighadeli S, Khosla R, Chu MT (2014). Socially assistive robots in elderly care: a mixed-method systematic literature review. Int J Hum Comput Interact.

[8] Abdi J, Al-Hindawi A, Ng T, Vizcaychipi MP (2018). Scoping review on the use of socially assistive robot technology in elderly care. BMJ Open.

[9] Huschilt J, Clune L (2012). The use of socially assistive robots for dementia care. J Gerontol Nurs.

[10] Marti P, Bacigalupo M, Giusti L, Mennecozzi C, Shibata T (2006). Socially assistive robotics in the treatment of behavioural and psychological symptoms of dementia. Proceedings of the 1st IEEE/RAS-EMBS International Conference on Biomedical Robotics and Biomechatronics.

[11] Bemelmans R, Gelderblom GJ, Jonker P, de Witte L (2012). Socially assistive robots in elderly care: a systematic review into effects and effectiveness. J Am Med Dir Assoc.

[12] Darragh M, Ahn HS, MacDonald B, Liang A, Peri K, Kerse N, Broadbent E (2017). Homecare robots to improve health and well-being in mild cognitive impairment and early stage dementia: results from a scoping study. J Am Med Dir Assoc.

[13] Vandemeulebroucke T, Dzi K, Gastmans C (2021). Older adults' experiences with and perceptions of the use of socially assistive robots in aged care: a systematic review of quantitative evidence. Arch Gerontol Geriatr.

[14] Abdi S, de Witte L, Hawley M (2020). Emerging technologies with potential care and support applications for older people: review of gray literature. JMIR Aging.

[15] Bharucha AJ, Anand V, Forlizzi J, Dew MA, Reynolds 3rd CF, Stevens S, Wactlar H (2009). Intelligent assistive technology applications to dementia care: current capabilities, limitations, and future challenges. Am J Geriatr Psychiatry.

[16] Rai HK, Cavalcanti Barroso A, Yates L, Schneider J, Orrell M (2020). Involvement of people with dementia in the development of technology-based interventions: narrative synthesis review and best practice guidelines. J Med Internet Res.

[17] Ienca M, Jotterand F, Vică C, Elger B (2016). Social and assistive robotics in dementia care: ethical recommendations for research and practice. Int J Soc Robot.

[18] Bedaf S, Marti P, Amirabdollahian F, de Witte L (2018). A multi-perspective evaluation of a service robot for seniors: the voice of different stakeholders. Disabil Rehabil Assist Technol.

[19] Seelye AM, Wild KV, Larimer N, Maxwell S, Kearns P, Kaye JA (2012). Reactions to a remote-controlled video-communication robot in seniors' homes: a pilot study of feasibility and acceptance. Telemed J E Health.

[20] Korchut A, Szklener S, Abdelnour C, Tantinya N, Hernández-Farigola J, Ribes JC, Skrobas U, Grabowska-Aleksandrowicz K, Szczęśniak-Stańczyk D, Rejdak K (2017). Challenges for service robots-requirements of elderly adults with cognitive impairments. Front Neurol.

[21] Wang S, Bolling K, Mao W, Reichstadt J, Jeste D, Kim HC, Nebeker C (2019). Technology to support aging in place: older adults' perspectives. Healthcare (Basel).

[22] Moyle W, Jones C, Cooke M, O'Dwyer S, Sung B, Drummond S (2014). Connecting the person with dementia and family: a feasibility study of a telepresence robot. BMC Geriatr.

[23] Boissy P, Corriveau H, Michaud F, Labonté D, Royer MP (2007). A qualitative study of in-home robotic telepresence for home care of community-living elderly subjects. J Telemed Telecare.

[24] Averill JB (2002). Matrix analysis as a complementary analytic strategy in qualitative inquiry. Qual Health Res.

[25] Miles MB, Huberman AM (1994). Qualitative Data Analysis: An Expanded Sourcebook. 2nd edition.

[26] Koceski S, Koceska N (2016). Evaluation of an assistive telepresence robot for elderly healthcare. J Med Syst.

[27] Park YH, Chang HK, Lee MH, Lee SH (2019). Community-dwelling older adults' needs and acceptance regarding the use of robot technology to assist with daily living performance. BMC Geriatr.

[28] Lai R, Tensil M, Kurz A, Lautenschlager NT, Diehl-Schmid J (2020). Perceived need and acceptability of an app to support activities of daily living in people with cognitive impairment and their carers: pilot survey study. JMIR Mhealth Uhealth.

[29] Law M, Sutherland C, Ahn HS, MacDonald BA, Peri K, Johanson DL, Vajsakovic DS, Kerse N, Broadbent E (2019). Developing assistive robots for people with mild cognitive impairment and mild dementia: a qualitative study with older adults and experts in aged care. BMJ Open.

[30] Wang RH, Sudhama A, Begum M, Huq R, Mihailidis A (2017). Robots to assist daily activities: views of older adults with Alzheimer's disease and their caregivers. Int Psychogeriatr.

[31] Pino M, Boulay M, Jouen F, Rigaud AS (2015). "Are we ready for robots that care for us?" Attitudes and opinions of older adults toward socially assistive robots. Front Aging Neurosci.

[32] Davis FD (1989). Perceived usefulness, perceived ease of use, and user acceptance of information technology. MIS Q.

[33] Wu YH, Wrobel J, Cornuet M, Kerhervé H, Damnée S, Rigaud AS (2014). Acceptance of an assistive robot in older adults: a mixed-method study of human-robot interaction over a 1-month period in the living lab setting. Clin Interv Aging.

[34] Verloo H, Kampel T, Vidal N, Pereira F (2020). Perceptions about technologies that help community-dwelling older adults remain at home: qualitative study. J Med Internet Res.

[35] Sriram V, Jenkinson C, Peters M (2019). Informal carers' experience of assistive technology use in dementia care at home: a systematic review. BMC Geriatr.

[36] Wu B (2020). Social isolation and loneliness among older adults in the context of COVID-19: a global challenge. Glob Health Res Policy.

[37] National Academies of Sciences, Engineering, and Medicine, Division of Behavioral and Social Sciences and Education, Health and Medicine Division, Board on Behavioral, Cognitive, and Sensory Sciences, Board on Health Sciences Policy, Committee on the Health and Medical Dimensions of Social Isolation and Loneliness in Older Adults (2020). Social Isolation and Loneliness in Older Adults: Opportunities for the Health Care System.

[38] Holt-Lunstad J, Smith TB, Layton JB (2010). Social relationships and mortality risk: a meta-analytic review. PLoS Med.

[39] Holt-Lunstad J (2020). Social isolation and health. Robert Wood Johnson Foundation.

[40] Daly JR, Depp C, Graham SA, Jeste DV, Kim HC, Lee EE, Nebeker C (2021). Health impacts of the stay-at-home order on community-dwelling older adults and how technologies may help: focus group study. JMIR Aging.

[41] Engelhart K (2021). What robots can- and can't-do for the old and lonely. The New Yorker.

